# Trends in Nonsurgical Interventions and Analgesic Medications for Low Back Pain: A Large Claims Database Study in Japan

**DOI:** 10.1111/papr.70080

**Published:** 2025-09-21

**Authors:** Kanako Makito, Asahi Fujita, Akira Honda, Akira Okada, Hideo Yasunaga

**Affiliations:** ^1^ Department of Biostatistics, School of Public Health The University of Tokyo Tokyo Japan; ^2^ Department of Ophthalmology, Graduate School of Medicine The University of Tokyo Tokyo Japan; ^3^ Department of Clinical Epidemiology and Health Economics, School of Public Health The University of Tokyo Tokyo Japan; ^4^ Department of Orthopaedic Surgery, Graduate School of Medicine Gunma University Maebashi Gunma Japan; ^5^ Department of Prevention of Diabetes and Lifestyle‐Related Diseases, Graduate School of Medicine The University of Tokyo Tokyo Japan

## Abstract

**Background:**

Low back pain is treated using a multidisciplinary strategy that includes several nonsurgical interventions and analgesic medications. However, trends in nonsurgical interventions and analgesic medications used in patients with low back pain remain unclear. This study aimed to investigate trends in nonsurgical interventions and analgesic medications among patients receiving nonsurgical interventions.

**Methods:**

We conducted a retrospective cohort study using the DeSC database (DeSC Healthcare Inc.), which contains health insurance claims and health checkup data of approximately 11 million patients in August 2021. We calculated the age‐ and sex‐adjusted rates of nonsurgical interventions and the proportions of analgesic medications used in patients who underwent nonsurgical interventions for chronic low back pain from 2015 to 2020. All trends were analyzed using the Cochran‐Armitage and Jonckheere‐Terpstra trend tests.

**Results:**

The rate of nonsurgical interventions tended to increase from 1.79 to 4.21 per 100 person‐years between 2015 and 2018 and declined slightly to 3.83 per 100 person‐years in 2020. Nonsurgical interventions, including spinal cord stimulation, demonstrated a significant increase (*p* = 0.01), whereas procedures such as epidurals did not show a statistically significant increase (*p* = 0.19). The age‐adjusted rate in females was higher than that in males after 2016. The sex‐adjusted rate in patients aged ≥ 60 years showed an increasing trend. Nonsteroidal anti‐inflammatory drugs were mostly used among patients receiving nonsurgical interventions; however, the proportion decreased significantly during the study period (*p* < 0.001). The proportion of patients using opioids was about 23%–30% and also decreased significantly (*p* < 0.001). The proportions of patients using acetaminophen, antiseizure medications, serotonin noradrenaline reuptake inhibitors, and tricyclic antidepressants increased significantly.

**Conclusion:**

The present study demonstrated an increasing trend in nonsurgical interventions, with a peak observed in 2018. Among them, interventions including spinal cord stimulation showed a significant upward trend, whereas interventions such as epidurals did not exhibit a statistically significant increase. There was a declining trend in the use of nonsteroidal anti‐inflammatory drugs and opioids among these patients. These findings may inform the selection of therapeutic interventions for managing low back pain.

## 1 Introduction

Chronic low back pain is a common disease that almost all adults experience during their lifetime and sometimes lasts for 12 weeks or more. The persistent pain limits their daily activities and causes a progressive disability [1, 2]. The treatments for chronic low back pain include nonpharmacologic, pharmacological, nonsurgical interventional, and surgical therapies, and multiple specialists are expected to provide a step‐by‐step approach or a combination of these treatments based on each patient's medical condition, personal needs, and specialist experience [3]. Minimally invasive procedures such as fluoroscopic neural block, lumbar radiofrequency (RF) ablation, lumbar pulsed RF, and spinal cord stimulation (SCS) have been developed using advanced neuroanatomic imaging and precise diagnostic and therapeutic injection techniques [4]. Some nonsurgical interventions are indicated for patients who do not respond well to noninvasive therapies, prefer surgical therapy, or are not suitable candidates for surgical therapy.

The prevalence of low back pain and the utilization of medical imaging, diagnostic tests, and therapies for low back pain have increased over the past decade [5, 6]. The use of epidural procedures increased by 7.3% annually from 2000 to 2009 but declined by 2.5% annually among Medicare enrollees from 2009 to 2018 [7]. Another study showed that the use and cost of nonsurgical interventional therapies such as lumbar RF increased from 2007 to 2016 in a commercially insured population in the United States [8]. In the Medicare population, the use of all types of facet joint interventions increased from 2000 to 2011 at a 13.6% average annual rate [9].

The efficacy of nonsurgical interventions for chronic low back pain is controversial [3], and they can be converted to surgical therapy and repeated due to the recurrence of pain and postsurgical pain in real‐world clinical practice. However, the trend of nonsurgical interventions and analgesic medications in patients who receive interventional pain management remains unclear in countries other than the United States.

In this study, we investigate the trends in nonsurgical interventions, including epidural and paravertebral blocks, SCS, and others (sympathetic blocks, selective nerve root blocks, sacroiliac joint blocks, lumbar facet joint blocks with or without RF, ethanol, or phenol injections), as well as the use of analgesic medications in patients receiving interventional pain treatment for chronic low back pain from 2015 to 2020, using a large administrative claims database in Japan.

## 2 Methods

### 2.1 Data Source

We used the DeSC database (DeSC Healthcare Inc.), which contains health insurance claims and health checkup data of approximately 11 million patients in August 2021. Since April 2015, the number of patients has increased annually. The database contains anonymous patient data collected from three health insurance systems: employees, non‐employees, and those aged ≥ 75 years [10].

The database contains information on the following patient data: age, sex, diagnoses, and comorbidities recorded as text data in the Japanese language and encoded with International Classification of Diseases and Related Health Problems, 10th Revision (ICD‐10) codes and Japanese original codes with dates of service; medical procedures encoded with Japanese original codes with dates of service; dispensed medications encoded with World Health Organization Anatomical Therapeutic Chemical (WHO‐ATC) codes and Japanese original codes with prescription date at physician visits; actual medical fees for the service of each patient; and medical checkup examination data.

Since this study relied on a secondary analysis of de‐identified administrative claims data, the requirement for informed consent was waived.

### 2.2 Outcomes

We identified patients who received epidural blocks, paravertebral blocks, SCS, and other nonsurgical interventions with or without RF, ethanol, or phenol injections (including sympathetic blocks, selective nerve root blocks, sacroiliac joint blocks, and facet joint blocks) between 2015 and 2020. We excluded patients < 20 years of age in each fiscal year and those with cancer‐related pain who were diagnosed with cancer (ICD‐10 code: C01‐09) and prescribed opioids were approved only for cancer‐related pain in Japan (WHO‐ATC code: N02AA, N02AA01, N02AA02, N02AA03, N02AA05, N02AA51, N02AB02, N02AB03, N02AB52, N02AD01, N02AE01, N02AX, N02AX06) before the date of nonsurgical interventions to identify patients with chronic low back pain. The outcome measures were the rates of nonsurgical interventions, which were categorized into the following two groups: (i) SCS and other nonsurgical interventions (sympathetic blocks, selective nerve root blocks, sacroiliac joint blocks, and facet joint blocks) with RF, ethanol, or phenol injections, and (ii) epidural blocks, paravertebral blocks, and other nonsurgical interventions (sympathetic blocks, selective nerve root blocks, sacroiliac joint blocks, and facet joint blocks) without RF, ethanol, or phenol injections. Additionally, we calculated the individual rates of spinal cord SCS, epidural blocks, paravertebral blocks, and other nonsurgical interventions, with and without RF, ethanol, or phenol injections. The numerator was the number of nonsurgical interventions, and the denominator was the person‐years of all beneficiaries aged ≥ 20 years. We subsequently stratified the population by the sex and age categories (20–39, 40–59, 60–79, ≥ 80 years old) and then calculated the rates of nonsurgical interventions in all the categories. The Japanese original codes for the aforementioned nonsurgical interventions are shown in Table S1.

We calculated the proportion of patients who underwent nonsurgical interventions and used analgesic medication in each fiscal year. The numerator was defined as the number of patients who received analgesic medication at least once. The medications associated with pain conditions were defined as follows: acetaminophen (N02BE01), nonsteroidal anti‐inflammatory drugs (NSAIDs) (M01AB, M01AB01, M01AB02, M01AB05, M01AB08, M01AB11, M01AB14, M01AC, M01AC01, M01AC05, M01AC06, M01AE, M01AE01, M01AE02, M01AE03, M01AE09, M01AE11, M01AE12, M01AG01, M01AG03, M01AH01, M01AX, M01AX01), antiseizure medications including pregabalin and mirogabalin (N02BG11, N03AX16), serotonin noradrenaline reuptake inhibitors (SNRI) (N06AX16, N06AX17, N06AX21), tricyclic antidepressants (TCA) (N06AA02, N06AA04, N06AA09, N06AA10), and tramadol or opioids which are approved for non‐cancer pain management in Japan (N02AA01, N02AA08, N02AA58, N02AB03, N02AE01, N02AJ, N02AX02).

### 2.3 Statistical Analysis

Person‐years were calculated for each fiscal year between 2015 and 2020. We calculated the rates of nonsurgical interventions per person‐year and the proportion of patients who received analgesic medications at least once among those who received nonsurgical interventions in each fiscal year. The rates of nonsurgical interventions were estimated using direct standardization for each year from 2015 to 2019, based on the age and sex distributions of the 2020 population. Direct standardization produces an adjusted rate, defined as the weighted sum of the crude rates, with the weights derived from the standard population distribution [11]. To investigate these trends, we applied the Cochran‐Armitage trend test for categorical data (proportions of medications) and the Jonckheere‐Terpstra trend test for continuous data (person‐years of nonsurgical interventions). All statistical analyses were performed using STATA/SE 18.1 (StataCorp, College Station, TX, USA).

## 3 Results

The number of patients aged ≥ 20 years in the DeSC database was 9,930,002 between April 2015 and March 2021. The median observation period was 2.92 years, and the total person‐years were 28,844,657 with an increase from 2,132,691 in 2015 to 6,994,712 in 2020. The proportion of patients aged ≥ 60 years increased gradually and accounted for > 50% of all patients since 2016 (Table 1).

**TABLE 1:** Person‐years of the health insurance beneficiaries and the number of nonsurgical interventions in each fiscal year.

	2015		2016		2017		2018		2019		2020		Total
Person‐years	2,132,691	(7.4)	3,591,907	(12.5)	4,070,722	(14.1)	5,802,267	(20.1)	6,252,358	(21.7)	6,994,712	(24.2)	28,844,657
Age, years													
20–39	508,532	(23.8)	601,466	(16.7)	662,264	(16.3)	696,117	(12.0)	736,261	(11.8)	756,146	(10.8)	
40–59	701,568	(32.9)	863,044	(24.0)	965,769	(23.7)	1,039,522	(17.9)	1,100,036	(17.6)	1,147,541	(16.4)	
60–79	819,886	(38.4)	1,572,596	(43.8)	1,863,215	(45.8)	2,731,224	(47.1)	3,018,311	(48.3)	3,401,480	(48.6)	
≥ 80	102,705	(4.8)	554,802	(15.4)	579,475	(14.2)	1,335,402	(23.0)	1,397,749	(22.4)	1,689,544	(24.2)	
Sex, male	1,051,349	(49.3)	1,708,050	(47.6)	1,942,225	(47.7)	2,656,449	(45.8)	2,862,442	(45.8)	3,186,832	(45.6)	
The number of beneficiaries	2,366,907		3,987,354		4,504,384		6,455,256		6,865,943		7,610,387		9,930,002
Age, years													
20–39	627,701	(26.5)	760,813	(19.1)	848,401	(18.8)	907,029	(14.1)	971,223	(14.1)	976,788	(12.8)	
40–59	764,728	(32.3)	960,867	(24.1)	1,068,741	(23.7)	1,166,588	(18.1)	1,229,020	(17.9)	1,281,008	(16.8)	
60–79	867,887	(36.7)	1,694,578	(42.5)	1,985,235	(44.1)	2,971,647	(46.0)	3,213,725	(46.8)	3,599,986	(47.3)	
≥ 80	106,591	(4.5)	571,096	(14.3)	602,007	(13.4)	1,409,992	(21.8)	1,451,975	(21.1)	1,752,605	(23.0)	
Sex, male	1,155,329	(48.8)	1,888,482	(47.4)	2,143,438	(47.6)	2,952,430	(45.7)	3,141,274	(45.8)	3,466,593	(45.6)	
The total number of nonsurgical interventions	29,964		86,079		94,088		237,302		243,122		264,644		
Age, years													
20–39	2292	(7.6)	2532	(2.9)	2955	(3.1)	3279	(1.4)	3519	(1.4)	3478	(1.3)	
40–59	9089	(30.3)	11,486	(13.3)	12,934	(13.7)	15,232	(6.4)	16,368	(6.7)	17,026	(6.4)	
60–79	16,221	(54.1)	44,992	(52.3)	50,518	(53.7)	112,695	(47.5)	119,920	(49.3)	128,242	(48.5)	
≥ 80	2362	(7.9)	27,069	(31.4)	27,681	(29.4)	106,096	(44.7)	104,527	(43.0)	117,228	(44.3)	
Sex, male	15,035	(50.2)	36,246	(42.1)	40,130	(42.7)	87,719	(37.0)	92,620	(38.1)	102,687	(38.8)	
The number of SCS and other nonsurgical interventions with RF, ethanol, or phenol injections	123		230		276		900		1212		1330		
Age, years													
20–39	14	(11.4)	10	(4.3)	16	(5.8)	5	(0.6)	14	(1.2)	22	(1.7)	
40–59	64	(52.0)	66	(28.7)	63	(22.8)	112	(12.4)	157	(13.0)	164	(12.3)	
60–79	40	(32.5)	110	(47.8)	158	(57.2)	522	(58.0)	684	(56.4)	768	(57.7)	
≥ 80	5	(4.1)	44	(19.1)	39	(14.1)	261	(29.0)	357	(29.5)	376	(28.3)	
Sex, male	58	(47.2)	125	(54.3)	135	(48.9)	422	(46.9)	593	(48.9)	598	(45.0)	
The number of epidural blocks, paravertebral blocks, and other nonsurgical interventions without RF, ethanol, or phenol injections	29,841		85,849		93,812		236,402		243,122		264,644		
Age, years													
20–39	2278	(7.6)	2522	(2.9)	2939	(3.1)	3274	(1.4)	3505	(1.4)	3456	(1.3)	
40–59	9025	(30.2)	11,420	(13.3)	12,871	(13.7)	15,120	(6.4)	16,211	(6.7)	16,862	(6.4)	
60–79	16,181	(54.2)	44,882	(52.3)	50,360	(53.7)	112,173	(47.5)	119,236	(49.0)	127,474	(48.2)	
≥ 80	2357	(7.9)	27,025	(31.5)	27,642	(29.5)	105,835	(44.8)	104,170	(42.8)	116,852	(44.2)	
Sex, male	14,977	(50.2)	36,121	(42.1)	39,995	(42.6)	87,297	(36.9)	92,027	(37.9)	102,089	(38.6)	

Abbreviations: RF, radiofrequency ablation; SCS, spinal cord stimulation.

**FIGURE 1:**
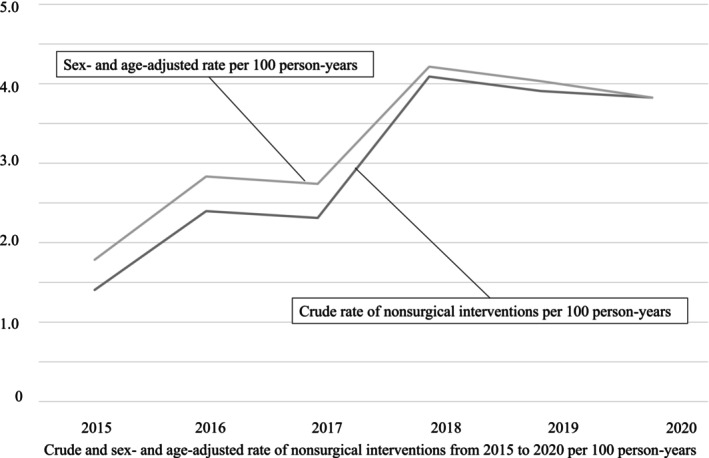
Crude and sex‐ and age‐adjusted rate of nonsurgical interventions from 2015 to 2020 per 100 person‐years.

**FIGURE 2:**
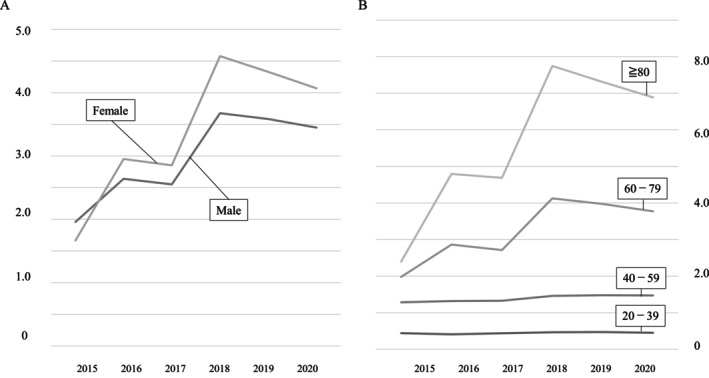
Sex‐ or age‐adjusted rate of nonsurgical interventions from 2015 to 2020 per 100 person‐years. (A) Age‐adjusted rates of nonsurgical interventions according to sex per 100 person‐years. (B) Sex‐adjusted rates of nonsurgical interventions by age category per 100 person‐years.

The sex‐ and age‐adjusted rates of nonsurgical interventions per 100 person‐years were 1.79 in 2015, 2.83 in 2016, 2.74 in 2017, 4.21 in 2018, 4.03 in 2019, 3.83 in 2020, with an increase of approximately 2.0% for 7 years, although not significantly (*p* = 0.19) and declined slightly after 2018 (Table 2 and Figure 1). The age‐adjusted rate of nonsurgical interventions of females per 100 person‐years was lower than that of males since 2016 (Figure 2A). The sex‐adjusted rates of nonsurgical interventions per 100 person‐years of patients aged ≥ 60 years rose and were higher than those of patients aged 20–59 years during the study period (Figure 2B).

**TABLE 2:** Trends in nonsurgical interventions between 2015 and 2020.

	2015	2016	2017	2018	2019	*2020*	*p*
Sex‐, age‐adjusted rate of all nonsurgical interventions per 100 person‐years	1.79	2.83	2.74	4.21	4.03	3.83	0.19
Sex‐adjusted rates per 100 person‐years							
Age, years							
20–39	0.44	0.41	0.44	0.46	0.47	0.45	0.19
40–59	1.28	1.32	1.33	1.46	1.48	1.47	0.01
60–79	1.98	2.86	2.71	4.13	3.97	3.77	0.19
≥ 80	2.39	4.79	4.69	7.74	7.31	6.89	0.19
Age‐adjusted rates per 100 person‐years							
Sex							
Male	1.96	2.64	2.55	3.68	3.58	3.45	0.19
Female	1.67	2.95	2.85	4.58	4.33	4.07	0.19
Sex‐, age‐adjusted rate of SCS and other nonsurgical interventions with RF, ethanol, or phenol injections (per 10,000 person‐years)	0.54	0.67	0.71	1.59	1.97	1.90	0.01
Sex‐, age‐adjusted rate of epidural blocks, paravertebral blocks, and nonsurgical interventions without RF, ethanol, or phenol injections (per 100 person‐years)	1.78	2.83	2.73	4.20	4.01	3.78	0.19

Abbreviations: RF, radiofrequency ablation; SCS, spinal cord stimulation.

**FIGURE 3:**
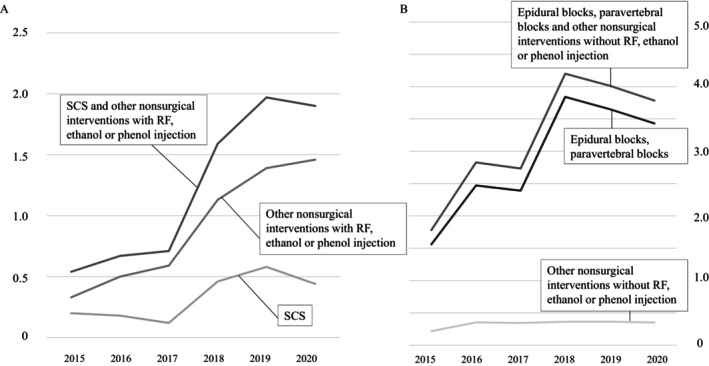
Sex‐ and age‐adjusted rate of SCS and nonsurgical interventions with RF, ethanol, or phenol injections, and epidural blocks or paravertebral blocks and other nonsurgical interventions without RF, ethanol, or phenol injections. (A): Sex‐ and age‐adjusted rates of SCS and nonsurgical interventions with RF, ethanol, or phenol injections per 10,000 person‐years. (B) Sex‐ and age‐adjusted rates of epidural blocks, paravertebral blocks, and other nonsurgical interventions without RF, ethanol, or phenol injections per 100 person‐years. RF, radiofrequency ablation; SCS, spinal cord stimulation.

The sex‐ and age‐adjusted rates of epidural blocks, paravertebral blocks, and other nonsurgical interventions without RF, ethanol, or phenol injections per 100 person‐years showed an increasing tendency, similar to that of the sex‐ and age‐adjusted rates of all nonsurgical interventions. The sex‐ and age‐adjusted rates of SCS and other nonsurgical interventions with RF, ethanol, or phenol injections per 10,000 person‐years also increased significantly (*p* = 0.01) (Table 2 and Figure 3A). The sex‐ and age‐adjusted rates of epidural and paravertebral blocks followed a similar trend to those of epidural blocks, paravertebral blocks, and other nonsurgical interventions without RF, ethanol, or phenol injections (Figure 3B).

**FIGURE 4:**
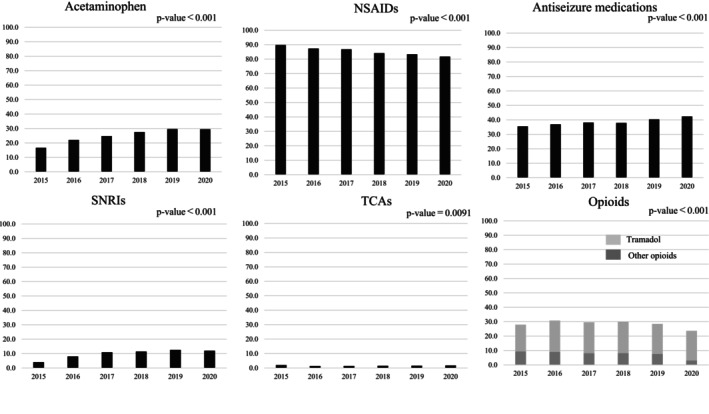
The proportions of analgesic medication use among patients receiving nonsurgical interventions by fiscal year.

Among the patients receiving nonsurgical interventions, the most frequently used analgesic medications were NSAIDs, and more than 80% of the patients used them at least once during the study period. Subsequently, antiseizure medications and opioids were used at least once by approximately 35%–45% and 23%–30% of the patients, respectively. The proportion of acetaminophen use, antiseizure medications, SNRIs, and TCAs increased significantly (all *p* < 0.05). In contrast, the proportion of NSAIDs and opioid use decreased significantly during the study period (all *p* < 0.05). The proportion of TCAs use was the lowest among the analgesic medications and ranged from 1.4% to 2.0% over 6 years (Figure 4).

## 4 Discussion

In the present study, we found that the trend of nonsurgical interventions for chronic low back pain tended to increase, although not significant, and declined after a peak in 2018. The age‐adjusted rates of males and females and the sex‐adjusted rates of patients aged ≥ 60 years showed increasing trends, whereas the sex‐adjusted rates of patients aged 20–59 years remained unchanged during the study period. The sex‐ and age‐adjusted rates of SCS and other nonsurgical interventions with RF, ethanol, or phenol injections significantly increased. However, the sex‐ and age‐adjusted rates of epidural blocks, paravertebral blocks, and other nonsurgical interventions without RF, ethanol, or phenol injections were likely to increase over the study period, although the difference was not significant.

Among the patients who received nonsurgical interventions, NSAIDs were prescribed most frequently but decreased significantly during the study period. Antiseizure medications and opioids were prescribed at least once to approximately 23%–45% of the patients. Prescriptions for acetaminophen, antiseizure medications, SNRIs, and TCAs tended to increase significantly.

A previous study that estimated low‐value care using the Japanese claims database showed that spinal injections for low back pain declined, while pregabalin prescriptions increased from 2015 to 2019 [12]. However, our results showed that the rate of nonsurgical interventions tended to increase. The inconsistency might be explained by the higher proportions of patients aged ≥ 60 years in the DeSC database compared with the Japanese age distribution. The proportion of patients aged ≥ 60 years in the DeSC database increased from 43.2% in 2015 to 72.8% in 2020, whereas the proportions of those aged ≥ 65 years were 26.6% in 2015 and 28.6% in 2020, according to the Japanese Population Census [13, 14]. Another possible reason is that the target population in the previous study was different from that in our study. The claims data in the previous study included information on patients in large acute care hospitals, while the data in the DeSC database included patients in general hospitals, clinics, and acute care hospitals. Additionally, we did not use the original Japanese procedure codes for trigger point injection and lumbar plexus block, which were included in patients with low back pain in a previous study.

The increasing tendency of SCS and other nonsurgical interventions with RF, ethanol, or phenol injections in the present study was similar to that reported in a previous study using United States commercial insurance claims [8]. A previous study suggested that one of the possibilities might be related to the opioid crisis in the United States, which did not occur in Japan because of the opioid prescription regulation for chronic pain management. In Japan, governmental guidelines regulate the appropriate use of medical opioids, restricting certain types, such as transdermal fentanyl, for non‐cancer pain. Additionally, only physicians who have completed certified e‐learning courses on non‐cancer pain management are authorized to prescribe these opioids [15].

The reasons for the increasing use of SCS and other nonsurgical interventions with RF, ethanol, or phenol injections remain unclear but can be attributed to multiple factors. These include the rising prevalence of the disease, patient characteristics, the incorporation of these techniques into multimodal pain management strategies, and clinical guidelines recommendations. One possible explanation is that older patients prefer less invasive treatments over surgical options due to concerns about surgical risks. Additionally, during the study period, RF was increasingly included in clinical practice guidelines as a minimally invasive technique for selected chronic spinal pain, supported by moderate to high‐quality evidence [4, 16, 17, 18]. Conversely, although anesthesiologists, particularly certified specialists, have traditionally been performing these procedures, their numbers have remained relatively stable, suggesting that this factor is unlikely to have influenced the observed trend [19]. The observed decrease in fiscal year 2020 may be attributed to the closure or reduction of medical services in many hospitals following the declaration of the COVID‐19 state of emergency.

Although not statistically significant, the rate of nonsurgical interventions, including epidural blocks, paravertebral blocks, and others without RF, ethanol, or phenol injections, increased until 2018, followed by a slight decline in subsequent years. This upward trend may be influenced by factors similar to those driving the increased use of SCS and other nonsurgical interventions with RF, ethanol, or phenol injections. These factors include the minimally invasive nature of the procedure, which provides rapid relief, supported by stable evidence [4, 16, 17, 18, 20]. Additionally, advancements in imaging technologies, such as improved ultrasound resolution, may have facilitated the expansion of interventional pain treatments in outpatient settings [21]. The decline observed after 2018 was primarily attributable to a reduction in the use of epidural and paravertebral blocks. Although the use of other nonsurgical interventions without RF, ethanol, or phenol injections, often performed for diagnostic purposes, did not increase, the trend may be partially explained by a growing preference for RF procedures. Further analyses using data from 2020 onward are warranted to clarify the underlying factors.

In this study, the most frequently used analgesic medications among patients who underwent nonsurgical intervention and did not have cancer‐related pain were NSAIDs, which are consistent with the recommendations of the guidelines [2, 17, 22, 23]. However, the use of NSAIDs significantly decreased, in line with findings from a previous study [24]. This may be attributed to the fact that NSAIDs have gastrointestinal, renal, and cardiovascular side effects, and the risk factors for complications need to be assessed, especially in older patients. In contrast, the proportion of acetaminophen significantly increased, which showed a similar trend to that in a previous study [24], because acetaminophen may have been prescribed more easily than NSAIDs, regardless of the side effects of NSAIDs. Both the 2012 low back pain guideline and the 2018 chronic pain guideline also recommended acetaminophen, alongside NSAIDs, as first‐line therapy for low back pain due to its high safety [17, 23]. Additionally, the dose of acetaminophen can be increased to a maximum of 4000 mg/day according to the patient's pain condition, which may contribute to the increased prescription of acetaminophen.

These findings contrast with those of a study investigating the epidemiology and medication use of patients with back pain presenting to emergency departments in the United States. That study reported opioids as the most commonly administered medications (40.7%), followed by acetaminophen (37.8%) and NSAIDs (22.6%) [25]. The observed discrepancies may be attributed to differences in patient characteristics and opioid prescription regulations between the United States and Japan.

Regarding trends in medications for pain management, previous studies from various countries have examined analgesic use, showing mixed trends depending on regional policies and clinical practice patterns [26, 27, 28]. In this study, the proportion of patients prescribed opioids, including tramadol, declined significantly. This trend contrasts with a previous finding, which showed a slight increase in opioid use for terminal cancer pain in Japan between 2010 and 2019 [29]. The lack of an increasing trend in opioid use may reflect differences in the number of opioids approved for cancer and non‐cancer pain, as well as cautious prescribing practices, especially for chronic non‐cancer pain, based on Japan's strict opioid regulations under the Narcotics and Psychotropics Control Act of 1953 [15]. The downward trend may also be influenced by the increasing availability of alternative pharmacological options. Although clinical guidelines and evidence do not always align with real‐world prescribing patterns, limited evidence on the long‐term efficacy and safety of opioids for chronic low back pain may also be contributing to this trend [17, 30]. The proportion of tramadol use accounted for over half of all opioids in our findings. This may be explained by the fact that tramadol prescriptions for non‐cancer pain management are not restricted to certified physicians, making it more readily accessible than other opioids.

The use of SNRIs in this study showed a significant upward trend, consistent with previous findings [24, 28]. The upward trend in SNRIs may primarily reflect the timing of sequential regulatory approvals for duloxetine in Japan. Duloxetine was initially approved for the treatment of major depressive disorder in 2010, followed by subsequent approvals for the treatment of pain associated with diabetic neuropathy in 2012, fibromyalgia in 2015, and chronic low back pain in 2016 [31]. Although the 2012 clinical guidelines acknowledged antidepressants' efficacy in managing chronic low back pain, their use was cautioned against due to the high incidence of side effects. However, SNRIs, particularly duloxetine, have been increasingly recognized for their efficacy in treating chronic low back pain and were reported to cause fewer side effects than TCAs [23, 32]. Following the approval of duloxetine for chronic low back pain, the 2018 Japanese guideline strongly recommended duloxetine for the treatment of musculoskeletal and neuropathic pain [17]. Similarly, the American College of Physicians guideline recommended duloxetine and tramadol as the second‐line medication for patients with chronic low back pain [22]. The background may have contributed to the observed increasing trend in SNRIs use during the study period.

In contrast, while TCAs use increased, it remained consistently lower than other analgesic medications. This may reflect their potential role as an alternative, though the trend suggests limited adoption in clinical practice. The prescription patterns of antidepressants in this study differed from a previous study, which showed amitriptyline as the most used antidepressant, followed by duloxetine [33]. While this may reflect regional differences in prescribing practices, the lower prescription rate may be due to concerns about side effects and the relatively limited evidence of efficacy for low back pain [17, 23, 32].

According to a systematic review and meta‐analysis of eight randomized controlled trials, pregabalin for the treatment of chronic low back pain was slightly less effective than other analgesics, including amitriptyline, celecoxib, and tramadol/acetaminophen [34]. A previous systematic review also showed that pregabalin was more effective than placebo in treating conditions such as post‐herpetic neuralgia, painful diabetic neuropathy, and mixed neuropathic pain [35]. The Japanese chronic pain practice guidelines strongly recommend the use of antiseizure medications for neuropathic pain but not for chronic lower back pain [17, 36]. However, antiseizure medications increased significantly and were used in approximately 35%–40% of the patients in this study, which is consistent with those of a previous study [12, 24]. The significant increase in antiseizure medication use, along with its higher prescription proportion compared to SNRIs and TCAs, may be associated with several factors. First, pregabalin was approved in 2010 for postherpetic neuralgia and peripheral neuropathic pain, and it became integrated into routine clinical practice earlier than duloxetine [37]. Second, in clinical settings, patients undergoing nonsurgical interventions for low back pain are often diagnosed with neuropathic pain. Although antidepressants have demonstrated efficacy for neuropathic pain, their use may be avoided due to potential side effects [17, 23].

A key strength of our study is its examination of treatment trends for low back pain in a real‐world clinical setting in Japan, where opioid prescriptions are relatively low compared to other countries [38]. This was achieved using a large‐scale database containing detailed information on medical procedures and medications. A previous study reported that interventional pain treatments for low back pain, including spinal injections and spinal fusion, represented the highest medical expenses in the fiscal year 2019, followed by pregabalin prescriptions [12]. Ideally, interdisciplinary pain management delivered by a team of physicians, pain‐informed psychologists, physical therapists, and other specialists could help reduce these medical expenses [39, 40].

The present study has some limitations. First, we could not distinguish the location of the spinal cord in the Japanese original codes for nonsurgical interventions. They included nonsurgical interventions for the cervical or thoracic spinal cord, and the number of nonsurgical interventions for chronic low back pain was overestimated. Second, some analgesic medications, such as NSAIDs, acetaminophen, SNRIs, and TCAs, may be misclassified because they are not always prescribed only for pain management. Third, the Japanese universal health insurance system limits the number of nonsurgical interventions per month; therefore, if more than a limited number of interventions were performed, the number might have been underestimated in this study.

## 5 Conclusions

Nonsurgical interventions demonstrated an increasing trend, peaking in 2018. Specifically, the use of interventions, including SCS, increased, whereas the use of NSAIDs and opioids decreased between 2015 and 2020 among patients receiving nonsurgical interventions for low back pain management. Although not statistically significant, the rate of nonsurgical interventions such as epidural blocks also showed a tendency to increase until 2018.

## Author Contributions

All listed authors should have contributed to the manuscript substantially and have agreed to the final submitted version. Kindly provide how all the authors have made a substantial contribution to the concept or design of the article; or the acquisition, analysis, or interpretation of data for the article.

## Ethics Statement

This study was approved by the Institutional Review Board of the University of Tokyo (IRB number: 2021010NI).

## Consent

Since this study relied on a secondary analysis of de‐identified administrative claims data, the requirement for informed consent was waived.

## Conflicts of Interest

The authors declare no conflicts of interest.

## Supporting information


**Table S1:** Japanese original codes of nonsurgical interventions.

## Data Availability

Data sharing is not applicable to this article as no new data were created or analyzed in this study.

## References

[1] J. M. Stevans , A. Delitto , S. S. Khoja , et al., “Risk Factors Associated With Transition From Acute to Chronic Low Back Pain in US Patients Seeking Primary Care,” JAMA Network Open 4 (2021): e2037371. 33591367 10.1001/jamanetworkopen.2020.37371 PMC7887659

[2] A. P. White , P. M. Arnold , D. C. Norvell , E. Ecker , and M. G. Fehlings , “Pharmacologic Management of Chronic Low Back Pain: Synthesis of the Evidence,” Spine (Phila Pa 1976) 36, no. Suppl (2011): S131–S143. 21952185 10.1097/BRS.0b013e31822f178f

[3] R. Chou , J. D. Loeser , D. K. Owens , et al., “Interventional Therapies, Surgery, and Interdisciplinary Rehabilitation for Low Back Pain: An Evidence‐Based Clinical Practice Guideline From the American Pain Society,” Spine (Phila Pa 1976) 34 (2009): 1066–1077. 19363457 10.1097/BRS.0b013e3181a1390d

[4] L. Manchikanti , S. Abdi , S. Atluri , et al., “An Update of Comprehensive Evidence‐Based Guidelines for Interventional Techniques in Chronic Spinal Pain. Part II: Guidance and Recommendations,” Pain Physician 16 (2013): S49–S283. 23615883

[5] B. I. Martin , R. A. Deyo , S. K. Mirza , et al., “Expenditures and Health Status Among Adults With Back and Neck Problems,” Journal of the American Medical Association 299 (2008): 656–664. 18270354 10.1001/jama.299.6.656

[6] B. I. Martin , J. A. Turner , S. K. Mirza , M. J. Lee , B. A. Comstock , and R. A. Deyo , “Trends in Health Care Expenditures, Utilization, and Health Status Among US Adults With Spine Problems, 1997‐2006,” Spine (Phila Pa 1976) 34 (2009): 2077–2084. 19675510 10.1097/BRS.0b013e3181b1fad1

[7] L. Manchikanti , M. R. Sanapati , A. Soin , et al., “An Updated Analysis of Utilization of Epidural Procedures in Managing Chronic Pain in the Medicare Population From 2000 to 2018,” Pain Physician 23 (2020): 111–126. 32214288

[8] J. B. Starr , L. Gold , Z. McCormick , P. Suri , and J. Friedly , “Trends in Lumbar Radiofrequency Ablation Utilization From 2007 to 2016,” Spine Journal 19 (2019): 1019–1028. 10.1016/j.spinee.2019.01.001 PMC6534485 30639589

[9] L. Manchikanti , V. Pampati , V. Singh , and F. J. Falco , “Assessment of the Escalating Growth of Facet Joint Interventions in the Medicare Population in the United States From 2000 to 2011,” Pain Physician 16 (2013): E365–E378. 23877460

[10] A. Okada and H. Yasunaga , “Prevalence of Noncommunicable Diseases in Japan Using a Newly Developed Administrative Claims Database Covering Young, Middle‐Aged, and Elderly People,” JMA Journal 5 (2022): 190–198. 35611228 10.31662/jmaj.2021-0189 PMC9090547

[11] T. L. Lash and K. J. Rothman , Modern Epidemiology, 4th ed. (Wolters Kluwer, 2021), 53–78. 10.1007/s10654-021-00778-w PMC8416883 34216355

[12] A. Miyawaki , R. Ikesu , Y. Tokuda , et al., “Prevalence and Changes of Low‐Value Care at Acute Care Hospitals: A Multicentre Observational Study in Japan,” BMJ Open 12 (2022): e063171. 10.1136/bmjopen-2022-063171 PMC9454035 36107742

[13] Statistics Bureau of Japan (SBJ) , Final Report of 2015 “Population and Households of Japan”, https://www.stat.go.jp/english/data/kokusei/2015/final_en/final_en.html.

[14] Statistics Bureau of Japan (SBJ) , “Summary of the Results and Statistical Tables,” (2020), https://www.stat.go.jp/english/data/kokusei/2020/summary.html.

[15] The Health, Labor and Welfare Ministry , Guidelines for the Appropriate Use of Medicinal Opioids, https://www.mhlw.go.jp/bunya/iyakuhin/yakubuturanyou/dl/iryo_tekisei_guide2017b.pdf.

[16] L. Manchikanti , M. V. Boswell , V. Singh , et al., “Comprehensive Evidence‐Based Guidelines for Interventional Techniques in the Management of Chronic Spinal Pain,” Pain Physician 12 (2009): 699–802. 19644537

[17] Japan Society of Pain Clinicians (JSPC) , Clinical Practice Guideline for the Management of Chronic Pain (2018), https://itami‐net.or.jp/nwp/cp‐bin/wordpress/wp‐content/uploads/2020/12/guideline‐e.pdf.

[18] Japan Society of Pain Clinicians (JSPC) , Guidelines for the Interventional Pain Treatment, https://jspc.gr.jp/Contents/public/kaiin_guideline02.html.

[19] Japan Society of Pain Clinicians (JSPC) , Trends in Membership and Designated Training Institutions, https://www.jspc.gr.jp/gaiyou/gaiyou?utm_source=chatgpt.com#&gid=1&pid=1.

[20] A. D. Kaye , L. Manchikanti , S. Abdi , et al., “Efficacy of Epidural Injections in Managing Chronic Spinal Pain: A Best Evidence Synthesis,” Pain Physician 18 (2015): E939–E1004. 26606031

[21] D. Viderman , M. Aubakirova , A. Aryngazin , et al., “Ultrasound‐Guided vs. Fluoroscopy‐Guided Interventions for Back Pain Management: A Systematic Review and Meta‐Analysis of Randomized Controlled Trials,” Diagnostics (Basel) 13 (2023): 3474. 37998610 10.3390/diagnostics13223474 PMC10670286

[22] A. Qaseem , T. J. Wilt , R. M. McLean , et al., “Noninvasive Treatments for Acute, Subacute, and Chronic Low Back Pain: A Clinical Practice Guideline From the American College of Physicians,” Annals of Internal Medicine 166 (2017): 514–530. 28192789 10.7326/M16-2367

[23] The Japanese Orthopaedic Association (JOA). JOA Clinical Practice Guideline for the Management of Low Back Pain (Nankodo, 2012).

[24] M. Akazawa , W. Mimura , K. Togo , et al., “Patterns of Drug Treatment in Patients With Osteoarthritis and Chronic Low Back Pain in Japan: A Retrospective Database Study,” Journal of Pain Research 12 (2019): 1631–1648. 31190973 10.2147/JPR.S203553 PMC6535438

[25] M. Gottlieb and K. Bernard , “Epidemiology of Back Pain Visits and Medication Usage Among United States Emergency Departments From 2016 to 2023,” American Journal of Emergency Medicine 82 (2024): 125–129. 38905718 10.1016/j.ajem.2024.06.020

[26] A. Hamina , A. E. Muller , T. Clausen , et al., “Prescription Opioids Among Older Adults: Ten Years of Data Across Five Countries,” BMC Geriatrics 22 (2022): 429. 35578167 10.1186/s12877-022-03125-0 PMC9112605

[27] C. Ju , L. Wei , K. K. C. Man , et al., “Global, Regional, and National Trends in Opioid Analgesic Consumption From 2015 to 2019: A Longitudinal Study,” Lancet Public Health 7 (2022): e335–e346. 35366408 10.1016/S2468-2667(22)00013-5

[28] L. R. Gorfinkel , D. Hasin , A. J. Saxon , et al., “Trends in Prescriptions for Non‐Opioid Pain Medications Among U.S. Adults With Moderate or Severe Pain, 2014‐2018,” Journal of Pain 23 (2022): 1187–1195. 35143969 10.1016/j.jpain.2022.01.006 PMC9271556

[29] R. Takahashi , M. Miyashita , Y. Murakami , and M. S. Oba , “Trends in Strong Opioid Prescription for Cancer Patients in Japan From 2010 to 2019: An Analysis With Large Medical Claims Data,” Japanese Journal of Clinical Oncology 52 (2022): 1297–1302, Erratum in: Jpn J Clin Oncol. 2023; 53:95. 35907780 10.1093/jjco/hyac122

[30] L. E. Chaparro , A. D. Furlan , A. Deshpande , A. Mailis‐Gagnon , S. Atlas , and D. C. Turk , “Opioids Compared to Placebo or Other Treatments for Chronic Low‐Back Pain,” Cochrane Database of Systematic Reviews 2013 (2013): CD004959. 23983011 10.1002/14651858.CD004959.pub4 PMC11056234

[31] The Health, Labor and Welfare Ministry , Report on the Deliberation Results, https://www.pmda.go.jp/files/000229930.pdf.

[32] R. Chou , R. Deyo , J. Friedly , et al., “Systemic Pharmacologic Therapies for Low Back Pain: A Systematic Review for an American College of Physicians Clinical Practice Guideline,” Annals of Internal Medicine 166 (2017): 480–492. 28192790 10.7326/M16-2458

[33] G. E. Ferreira , M. di Donato , C. G. Maher , C. A. Shaheed , S. Mathieson , and A. Collie , “Patterns of Antidepressant Use in People With Low Back Pain: A Retrospective Study Using Workers' Compensation Data,” European Journal of Pain 29 (2025): e4773. 39688137 10.1002/ejp.4773

[34] H. Shanthanna , I. Gilron , M. Rajarathinam , et al., “Benefits and Safety of Gabapentinoids in Chronic Low Back Pain: A Systematic Review and Meta‐Analysis of Randomized Controlled Trials,” PLoS Medicine 14 (2017): e1002369. 28809936 10.1371/journal.pmed.1002369 PMC5557428

[35] S. Derry , R. F. Bell , S. Straube , P. J. Wiffen , D. Aldington , and R. A. Moore , “Pregabalin for Neuropathic Pain in Adults,” Cochrane Database of Systematic Reviews 1 (2019): CD007076. 30673120 10.1002/14651858.CD007076.pub3 PMC6353204

[36] Japan Society of Pain Clinicians (JSPC) , Guidelines for the Pharmacologic Management of Neuropathic Pain, Second Edition, https://www.jspc.gr.jp/Contents/public/kaiin_guideline06.html.

[37] Pfizer Inc ., “Pfizer's Lyrica® (Pregabalin) Capsules CV Receives Approval for Treatment of Peripheral Neuropathic Pain In Japan,” (2010), https://www.pfizer.com/news/press‐release/press‐release‐detail/pfizer_s_lyrica_pregabalin_capsules_cv_receives_approval_for_treatment_of_peripheral_neuropathic_pain_in_japan.

[38] T. Miyachi , A. Ozaki , H. Saito , T. Sawano , T. Tanimoto , and A. Crump , “Opioids: A ‘Crisis’ of Too Much or Not Enough – Or Simply How Rich You Are and Where You Live?,” European Journal of Pain 25 (2021): 1181–1194. 33822443 10.1002/ejp.1767

[39] B. K. Frogner , K. Harwood , C. H. A. Andrilla , M. Schwartz , and J. M. Pines , “Physical Therapy as the First Point of Care to Treat Low Back Pain: An Instrumental Variables Approach to Estimate Impact on Opioid Prescription, Health Care Utilization, and Costs,” Health Services Research 53 (2018): 4629–4646. 29790166 10.1111/1475-6773.12984 PMC6232429

[40] P. M. Herman , M. L. Anderson , K. J. Sherman , B. H. Balderson , J. A. Turner , and D. C. Cherkin , “Cost‐Effectiveness of Mindfulness‐Based Stress Reduction Versus Cognitive Behavioral Therapy or Usual Care Among Adults With Chronic Low Back Pain,” Spine (Phila Pa 1976) 42 (2017): 1511–1520. 28742756 10.1097/BRS.0000000000002344 PMC5694631

