# Supplementary material for: Trends in Nonsurgical Interventions and Analgesic Medications for Low Back Pain: A Large Claims Database Study in Japan
Source: Pain Pract. 2025 Sep 21;25(8):e70080. doi: 10.1111/papr.70080 (PMC12451095; doi:10.1111/papr.70080)
Supplement: Supplementary file 1 — Table S1: Japanese original codes of nonsurgical interventions. [file PAPR-25-0-s001.docx]

Supplementary Table 1. Japanese original codes of nonsurgical interventions

| 150265710, 150241810 | Selective nerve root blocks with RF, Ethanol, or Phenol injections |
| --- | --- |
| 150351610 | Sacroiliac joint blocks with RF, Ethanol, or Phenol injections |
| 150351710, 150239210 | Facet joint blocks with RF, Ethanol, or Phenol injections |
| 150239110 | Thoracic or lumbar sympathetic nerve blocks with RF, Ethanol or Phenol injections |
| 150255210, 150384810, 150398010, 150398110, 150383050 | Spinal cord stimulation |
| 150242110 | Epidural blocks |
| 150235510 | Lumbar epidural blocks |
| 150236010 | Caudal epidural blocks |
| 150237710 | Paravertebral blocks |
| 150265010, 150238510 | Selective nerve root blocks without RF, Ethanol, or Phenol injections |
| 150351010 | Sacroiliac joint blocks without RF, Ethanol, or Phenol injections |
| 150351110 | Facet joint blocks without RF, Ethanol, or Phenol injections |
| 150235710 | Lumbar sympathetic blocks without RF, Ethanol, or Phenol injections |
